# B-Myb deficiency boosts bortezomib-induced immunogenic cell death in colorectal cancer

**DOI:** 10.1038/s41598-024-58424-w

**Published:** 2024-04-02

**Authors:** Yuan-Jian Hui, Ting-Ting Yu, Liu-Gen Li, Xing-Chun Peng, Mao-Jun Di, Hui Liu, Wen-Long Gu, Tong-Fei Li, Kai-Liang Zhao, Wei-Xing Wang

**Affiliations:** 1 Department of Hepatobiliary Surgery, Renmin Hospital of Wuhan University, Jiefang Road No. 238, Wuhan, 430060 Hubei Province China; 2 Department of General Surgery, Taihe Hospital, Hubei University of Medicine, Renmin South Road No. 32, Shiyan, 442000 Hubei Province China; 3 Hubei Key Laboratory of Embryonic Stem Cell Research, School of Basic Medical Sciences, Hubei University of Medicine, Renmin South Road No. 30, Shiyan, 442000 Hubei Province China; 4 Department of Pathology, Renmin Hospital of Shiyan, Hubei University of Medicine, Shiyan, 442000 Hubei Province China

**Keywords:** B-Myb (MYBL2), Bortezomib (BTZ), DNA damage, Immunogenic death (ICD), Macrophages, Bioinformatics, Cancer, Drug discovery

## Abstract

B-Myb has received considerable attention for its critical tumorigenic function of supporting DNA repair. However, its modulatory effects on chemotherapy and immunotherapy have rarely been reported in colorectal cancer. Bortezomib (BTZ) is a novel compound with chemotherapeutic and immunotherapeutic effects, but it fails to work in colorectal cancer with high B-Myb expression. The present study was designed to investigate whether B-Myb deletion in colorectal cancer could potentiate the immune efficacy of BTZ against colorectal cancer and to clarify the underlying mechanism. Stable B-Myb knockdown was induced in colorectal cancer cells, which increased apoptosis of the cancer cells relative to the control group in vitro and in vivo. We found that BTZ exhibited more favourable efficacy in B-Myb–defective colorectal cancer cells and tumor-bearing mice. BTZ treatment led to differential expression of genes enriched in the p53 signaling pathway promoted more powerful downstream DNA damage, and arrested cell cycle in B-Myb–defective colorectal cancer. In contrast, recovery of B-Myb in B-Myb–defective colorectal cancer cells abated BTZ-related DNA damage, cell cycle arrest, and anticancer efficacy. Moreover, BTZ promoted DNA damage–associated enhancement of immunogenicity, as indicated by potentiated expression of HMGB1 and HSP90 in B-Myb–defective cells, thereby driving M1 polarization of macrophages. Collectively, B-Myb deletion in colorectal cancer facilitates the immunogenic death of cancer cells, thereby further promoting the immune efficacy of BTZ by amplifying DNA damage. The present work provides an effective molecular target for colorectal cancer immunotherapy with BTZ.

## Introduction

Colorectal cancer is the most common malignant tumor of the digestive system, and it seriously affects patients’ quality of life^1,2^. Surgery is the primary treatment option for colorectal cancer, but the recurrence rate is as high as 30%^3^. Therefore, chemotherapy combined with surgery has become the current dominant strategy in the treatment of colorectal cancer^4–6^. Most chemotherapeutic agents have toxic side effects on the normal tissues and cells of the body. Bortezomib (BTZ) is a novel compound with both chemotherapeutic and immunotherapeutic effects. It has recently been approved by the US Food and Drug Administration for a variety of solid tumors^7^. Its primary mechanism of action is based on the inhibition of the intracellular proteasome, and BTZ has few toxic side effects on normal cells^8,9^. However, many studies have revealed that BTZ is often ineffective against malignant tumors due to drug resistance, which greatly limits its clinical application^10–12^.

The MBY gene family, MYBL2, also known as B-Myb, is highly expressed in several solid tumors, including breast cancer, non–small-cell lung cancer, and colorectal cancer^13–16^. B-Myb plays a promotive role in tumor development, and its functions are mostly related to apoptosis, differentiation, and proliferation^17–19^. It has been suggested that B-Myb can boost cell proliferation by encouraging the expression of cell cycle–dependent kinase (CDK) and cell cycle–related proteins^20,21^. B-Myb also supports the DNA repair response (DDR) after damage. Therefore, a higher level of B-Myb limits DNA damage caused by chemotherapeutic agents^22^. Therefore, we hypothesized that reduced expression of B-Myb in colorectal cancer could effectively attenuate the DDR, thereby promoting BTZ-mediated DNA damage and release of DNA damage–associated molecular patterns (DAMPs), leading to immunogenic cell death of the cancer cells. The increased expression and release of major nucleus-derived DAMPs, including HMGB1 and HSP90, can augment the local inflammatory response, thereby promoting the infiltration of macrophages into the tumor’s periphery and their polarization to the M1 phenotype. The mechanism of resistance to BTZ is still unclear. Most studies have focused on BTZ-induced ubiquitination of intracellular proteins^23–26^. However, there is also evidence suggesting that DNA damage and immune microenvironment activation are involved in chemotherapeutic drug resistance. Based on this, we examined whether BTZ treatment promotes the immunogenic cell death of colorectal cancer cells by stimulating DNA damage. We also investigated whether deletion of B-Myb impairs DNA repair and further promotes BTZ-induced apoptosis.

Herein, the B-Myb of colorectal cancer cells was stably knocked down, which increased the apoptosis levels of the cancer cells. Then, the anticancer effect of BTZ was investigated in B-Myb–deficient cells and control cells, and the in vivo efficacy was also evaluated. We found that BTZ amplified DNA damage and arrested the cell cycle in B-Myb–deleted cancer cells. In contrast, the recovery of B-Myb abated the BTZ-mediated enhanced anticancer efficacy in B-Myb–deficient cells, proving that B-Myb reversed the anticancer effect of BTZ. Most importantly, BTZ led to DNA damage–associated enhancement of immunogenicity, as indicated by the potentiated expression of HMGB1 and HSP90 in B-Myb–defective colorectal cancer, further driving M1 polarization of macrophages. Based on our results, B-Myb may be an effective molecular target for colorectal cancer immunotherapy with BTZ.

## Materials and methods

### Cell lines and culture

SW480 and SW620 colorectal cancer cells were purchased from the Cell Bank of Shanghai Institutes for Biological Sciences (Shanghai, China). Stable B-Myb knockdown was induced in these cells by lentiviral shRNA (B-Myb). The stable cell clones expressing the shRNA constructs were isolated using puromycin. The control SW480 cells were named shNC, and the lentiviral shRNA–treated SW480 cells were named shB-Myb. All of the cells were cultured in Dulbecco's Modified Eagle Medium (Sigma-Aldrich, St Louis, USA) supplemented with 10% fetal bovine serum (QmSuero/Tsingmu Biotechnology, Wuhan) in an incubator with humidification (5% CO_2_/95% air) at 37 °C.

### Tumor-bearing mouse model

Female BALB/c-nu mice at 4 weeks of age (16–18 g) were kept at the Animal Center at the Hubei University of Medicine (Shiyan, China) in a temperature-controlled environment and were fed a rodent diet. The animal handling and experimental procedures conformed to the protocols approved by the Animal Care Committee at the Hubei University of Medicine. All inoculations and treatments were carried out under pentobarbital (Nembutal) anesthesia. To construct a colorectal cancer–bearing mouse model, the SW480 cells of shNC and shB-Myb were subcutaneously inoculated into the axilla of the mice (subcutaneous xenograft). For about 10 days, mice were randomly divided into four groups (4 per group). BTZ treatment (i.p., 1 mg/kg, every three days for a total of 5 times) was administrated when the tumor grew to 150–200 mm^3^. Although lymphocyte counts were reduced in BALB/c-nu mice, macrophage infiltration in the tumor grafts was not affected. We confirmed that all methods were performed in accordance with the relevant guidelines and regulations of the Animal Care Committee at the Hubei University of Medicine.

### Bioinformatic analysis

To analyze the expression and clinical relevance of B-Myb (MYBL2) in pan-cancer and colorectal cancer, the Gene Expression Profiling Interactive Analysis (GEPIA2) and Tumor IMmune Estimation Resource (TIMER) websites (http://gepia2.cancer-pku.cn/, https://cistrome.shinyapps.iotimer/) were utilized. The expression and clinical prognosis of B-Myb in colorectal adenocarcinoma (COAD) and rectum adenocarcinoma (READ) were analyzed and presented.

### *Bortezomib treatment *in vitro* and *in vivo

For the treatment of SW480 cells in vitro, we first screened the effective concentrations and incubation time of BTZ. The cell viability and DNA damage was assayed using CCK-8 and comet assay at different concentrations of BTZ. In the following experiments, the cells were treated with BTZ (30 nM) for 24 h and used to conduct various measurements except for special explanations. The levels of apoptosis and proliferation were detected by western blot (WB) and propidium iodide (PI) staining. To investigate the in vivo efficacy, BTZ (1 mg/kg) was injected (i.p.) into the mice once every 3 days for a total of 5 doses. At 24 h after the last treatment, the mice were sacrificed and the tumor grafts were harvested for analysis.

### Apoptosis assay of cancer cells

To detect the level of apoptosis of colorectal cancer cells, the proteins of shNC- and shB-Myb-treated cells were extracted. Then, the apoptosis- and proliferation-associated molecules Bax, Caspase-3, and PCNA were detected by WB. Alternatively, the cells were stained using PI and observed under laser confocal scanning microscopy (LCSM). In addition, PI-positive cells were counted by flow cytometry. The resulting sticky 3'-OH termini were measured by TUNEL staining (C1088, Beyotime, Shanghai, China).

### RNA sequencing and data analysis

The concentration and purity of RNA were measured using NanoDrop 2000, and the integrity of RNA was assessed using the RNA Nano 6000 Assay Kit of the Agilent Bioanalyzer 2100 system. A total amount of 1 μg RNA per sample was used as input material for the RNA sample preparations. Sequencing libraries were generated using the NEBNext UltraTM RNA Library Prep Kit for Illumina (NEB, USA) following the manufacturer’s recommendations, and index codes were added to attribute sequences to each sample. Differential expression of genes and KEGG pathway enrichment were analyzed from the raw data with a bioinformatic pipeline tool, BMKCloud (www.biocloud.net) online platform.

### DNA damage measurement

The DNA damage–associated molecules γ-H2A.X and p53 were detected with WB. The direct DNA damage, also known as DNA double-strand breakage (DDSB), was assayed by comet assay. Briefly, the cells were seeded in 24-plates and treated with BTZ. The cell suspensions were prepared and mixed with low-melting-point agarose. The mixture was then dripped onto a glass slide pre-coated with agarose gel and pressed, followed by electrophoresis at 25 V and 250 mA for 18 min. The mixture was then neutralized using tris–Hcl (pH = 6.0) for 30 min. Finally, Hoechst 33,342 was used to stain the nuclei. The cells with tails were photographed using fluorescence microscopy.

### Cell cycle and immunogenic assays

The molecules involved in the cell cycle (CDK4, CDC25A, and CyclinD1) were analyzed by WB. The cells treated with BTZ were obtained and washed in phosphate-buffered saline (PBS). The cells were fixed and labeled with PI after the RNA had been removed by RNAase. The labeled cells were washed three times and harvested with flow cytometry. The cells in the G1, S, and G2/M phases were counted separately. The DNA damage–associated immunogenic molecules (HMGB1 and HSP90) were analyzed using WB.

### Overexpression of B-Myb

The cells with stable knockdown of B-Myb were seeded in 24-well or 6-well plates and incubated with B-Myb–overexpressing plasmid and Lipofectamine 3000 (1:1 at volume) for about 8 h. The Lipofectamine 3000- and plasmid-containing medium was replaced by a fresh medium after the transfection. The successful overexpression of B-Myb was confirmed by the increased expression of B-Myb detected by WB in B-Myb–deleted cells. The transfected cells were then treated with BTZ. The DNA damage, cell cycle, and apoptosis were assayed.

### Detection of anticancer efficacy in vivo

To detect the anticancer efficacy of BTZ in vivo, the tumor-bearing mice were obtained as described before. For about 10 days, BTZ treatment was initiated when the tumor grew to 150 mm^3^. BTZ was administered every 3 days for a total of five doses. At 24 h after the last treatment, the mice were sacrificed and the tumor grafts were harvested. The tumor volume was monitored during the treatment. The tumor tissues were weighted after the extraction of the tumor grafts and the tumor grafts were photographed. TUNEL staining was utilized to detect apoptosis of the cancer cells in the tumor grafts. For the detection of apoptosis–associated molecules (Caspase-3 and Bax), paraffin-embedded sections of the neoplastic tissue were prepared for immunohistochemistry (IHC).

### IHC and double immunofluorescence staining assay in tumor-bearing mice

The paraffin-embedded sections of the neoplastic tissue were dewaxed, rehydrated, and antigen-repaired with sodium citrate for 30 min. Then, they were incubated with 3% hydrogen peroxide at room temperature for 20 min. Next, the sections were blocked with 3% BSA for 1 h and labeled with a primary antibody at 4 °C overnight. The following primary antibodies were used: CDK4 (11026-1-AP, Proteintech, Wuhan, China), CydlinD1 (26939-1-AP, Proteintech, Wuhan, China), CDC25A (55031-1-AP, Proteintech, Wuhan, China), Bax (bs-0127R, Bioss, Beijing, China), Caspase-3 (50599-2-Ig, Proteintech, Wuhan, China), PCNA (bs-2006R, Bioss, Beijing, China), Ki67 (bs-23103R, Bioss, Beijing, China), B-Myb (ab12296, Abcam, Cambridge, United Kingdom), γ-H2A.X (bs-3185R. Bioss, Beijing, China), and p53 (bs-2090R, Bioss, Beijing, China). The sections were stained with iNOS, GBP5, and CD11b to analyze the phenotype of macrophages. The samples were subsequently stained with a secondary antibody (PV-9000, ZSGB-BIO, Beijing, China) at 37 °C for 1 h. Diaminobenzidine (DAB, ZL-9018, ZSGB-BIO, Beijing, China) was used for staining at room temperature for 1–3 min. The nuclei were stained with hematoxylin or DAPI. Finally, the paraffin-embedded sections were observed with an orthogonal Olympus microscope or a confocal microscope.

### Flow cytometry detection

The excitation and emission wavelengths in the FITC channel were 488 and 525 nm, respectively, and those in the PE channel were 561 and 585 nm, respectively. After the cells were processed and collected as described above, they were filtered into special tubes for flow cytometry. Each channel was adjusted to the appropriate voltage before collecting the cells. At least 1 × 10^4^ cells per sample were acquired for every collection. Geometric means (GM) were used to quantify the mean fluorescent intensity (MFI).

### Western blotting

The cells treated as described before were lysed in a RIPA buffer containing a protease inhibitor for 60 min. The cell lysates were centrifuged, and their protein concentration was measured using a bicinchoninic acid (BCA) assay kit. Equal amounts of protein (10–30 μg) were fractionated by SDS-PAGE and transferred to PVDF membranes. The membranes were blocked with 3–5% BSA in TBST and incubated with primary antibodies against CDK4 (11026-1-AP, Proteintech, Wuhan, China), CydlinD1 (26939-1-AP, Proteintech, Wuhan, China), CDC25A (55031-1-AP, Proteintech, Wuhan, China), Bax (bs-0127R, Bioss, Beijing, China), Caspase-3 (50599-2-Ig, Proteintech, Wuhan, China), PCNA (bs-2006R, Bioss, Beijing, China), Ki67 (bs-23103R, Bioss, Beijing, China), B-Myb (ab12296, Abcam, Cambridge, United Kingdom), γ-H2A.X (bs-3185R. Bioss, Beijing, China), p53 (bs-2090R, Bioss, Beijing, China), GBP5 (13220-1-AP, Proteintech, Wuhan, China), iNOS (ab15323, Abcam, Cambridge, UK), and GAPDH (PMK053C, BioPM, Wuhan, China) at 4 °C overnight. Then, the membranes were incubated with horseradish peroxidase–conjugated secondary antibody. Finally, the bands were incubated using an ECL kit (PMK003, BioPM, Wuhan, China) and exposed using a Bio-Imaging system (170–8265, Bio-Rad). The result of gels images was cropped and original blots with membrane edges visible were included in the Supplementary Materials (Figs. S9 and S10). Regarding the absence of original blots of adequate length, we provided the following explanations: (1). Because there are molecular weight differences between some of the proteins, the membranes were cut prior to hybridisation with antibodies to ensure that each protein could be properly exposed. (2). We harnessed the Bio-Rad imaging system for blot scanning, the scanning settings caused the blots’ edges being smaller than the original bands, leading to the partial bands’ edge regions not to be fully displayed. We assure that the samples derive from the same experiment.

### Statistical analysis

The data were presented as the mean ± standard deviation (SD). The data was processed and statistically analyzed using Graphpad Prism 8.0. software. Statistical differences between groups were analyzed by one-way analysis of variance (ANOVA) or Student’s *t* text. *p* < 0.05 were considered statistically significant. For image processing and the presentation of statistical results, Photoshop sofware was harnessed.

### Ethics approval and consent to participate

Animal handling and experimental procedures conformed to the protocols approved by the Animal Care Committee at the Hubei University of Medicine (Approval Number: 2022. No. 66). The Animal Care Committee at the Hubei University of Medicine specifies that the tumor size in tumor-bearing mice should generally not exceed 2000 mm^3^. We confirmed that all methods were performed in accordance with the relevant guidelines and regulations.

## Results

### Downregulation of B-Myb facilitates apoptosis of colorectal cancer

**Figure 1:**
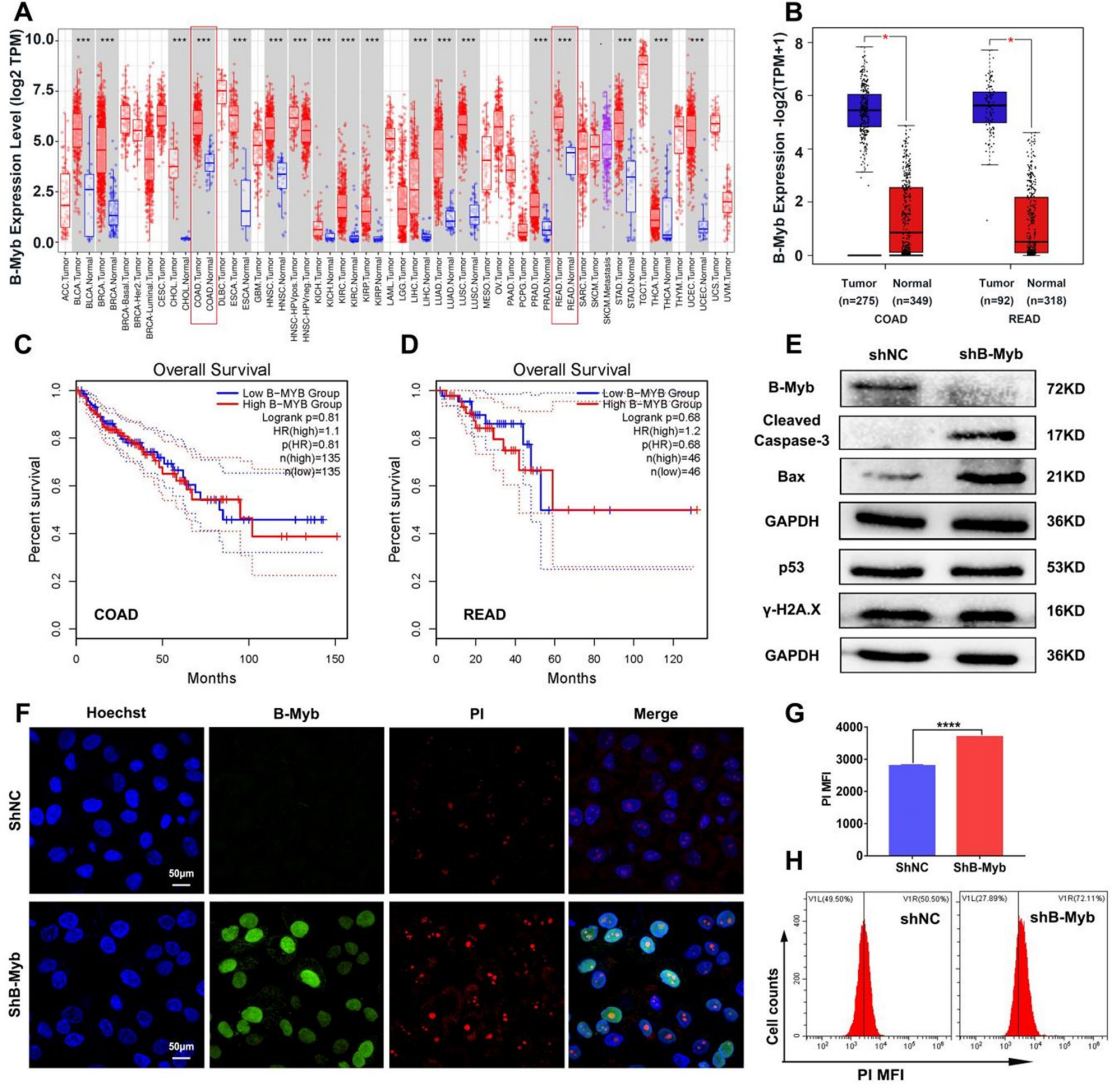
Stable knockdown of B-Myb led to increased apoptosis in colorectal cancer cells. (**A–B**) The B-Myb (MYBL2) expression in pan-cancer, COAD and READ was analyzed by bioinformatics. (**C–D**) The prognostic correlation between the expression of B-Myb was presented. The SW480 cells with or without B-Myb knockdown were cultured for 24 h. (**E**) The expression of apoptosis proteins Bax, Caspase-3 and B-Myb was detected by WB, which was also utilized to analyze the DNA damage-associated molecules p53 and γ-H2A.X expression. (**F–H**) The apoptosis of SW480 cells was measured through PI staining. The PI positive cells were detected with confocal microscopy (**F**) and flow cytometry (**G**–**H**). Geometric means were used to quantify the MFI. Values were means ± SD (n = 3, ****p < 0.0001).

First, the expression levels of B-Myb in pan-cancer and colorectal cancer were analyzed using bioinformatics. We found that B-Myb was significantly upregulated in cancer tissues compared with normal tissues (Fig. 1A–B), although which was not associated with overall survival in COAD and READ as presented in Fig. 1C–D. We further validated for high B-Myb in SW480 and SW620 as experimental candidates and constructed cell lines with stable knockdown of B-Myb (Figs. 1E and S1). To explore how the deficit of B-Myb affects the apoptosis level of colorectal cancer cells, we first examined the direct apoptosis rate in B-Myb–stable-knockdown cells and control cells. As displayed in Figs. 1E and S1, the decreased protein expression of B-Myb in the sh-B-Myb group indicated the successful construction of the stable knockdown model of B-Myb in colorectal cancer cells. Apoptosis appeared in B-Myb–deficient colorectal cancer cells, as evidenced by the enhanced expression of cleaved-caspase-3 and Bax (Fig. 1E). Furthermore, PI staining revealed prominent cell death following B-Myb downregulation (Fig. 1F–H). Nevertheless, the expression of DNA damage–associated molecules (p53 and γ-H2A.X) varied little with the downregulation of B-Myb (Fig. 1E). Consistently, the in vivo findings suggested that the tumor grafts were prone to apoptosis, given the elevated Bax and Caspase-3 expression in the tumor tissues of the sh-B-Myb cancer cell–bearing mice (Fig. S2A–C). Moreover, TUNEL staining revealed potentiated apoptosis of the cancer cells in the tumor grafts of the shB-Myb cancer cells–bearing mice (Fig. S2D). Nevertheless, the absence of B-Myb did not affect the expression levels of p53, γ-H2A.X, HMGB1, and HSP90, suggesting the lack of apparent DNA damage and DAMPs release in B-Myb–deficient tumor tissues (Figs. S2E–F, S3, S4). In summary, lack of B-Myb promoted apoptosis of colorectal cancer cells slightly. We speculated that B-Myb deletion could activate the anticancer effect of chemotherapeutic agents.

### BTZ exhibits pronounced anticancer efficacy in B-Myb–deficient colorectal cancer cells and tumor tissues

**Figure 2:**
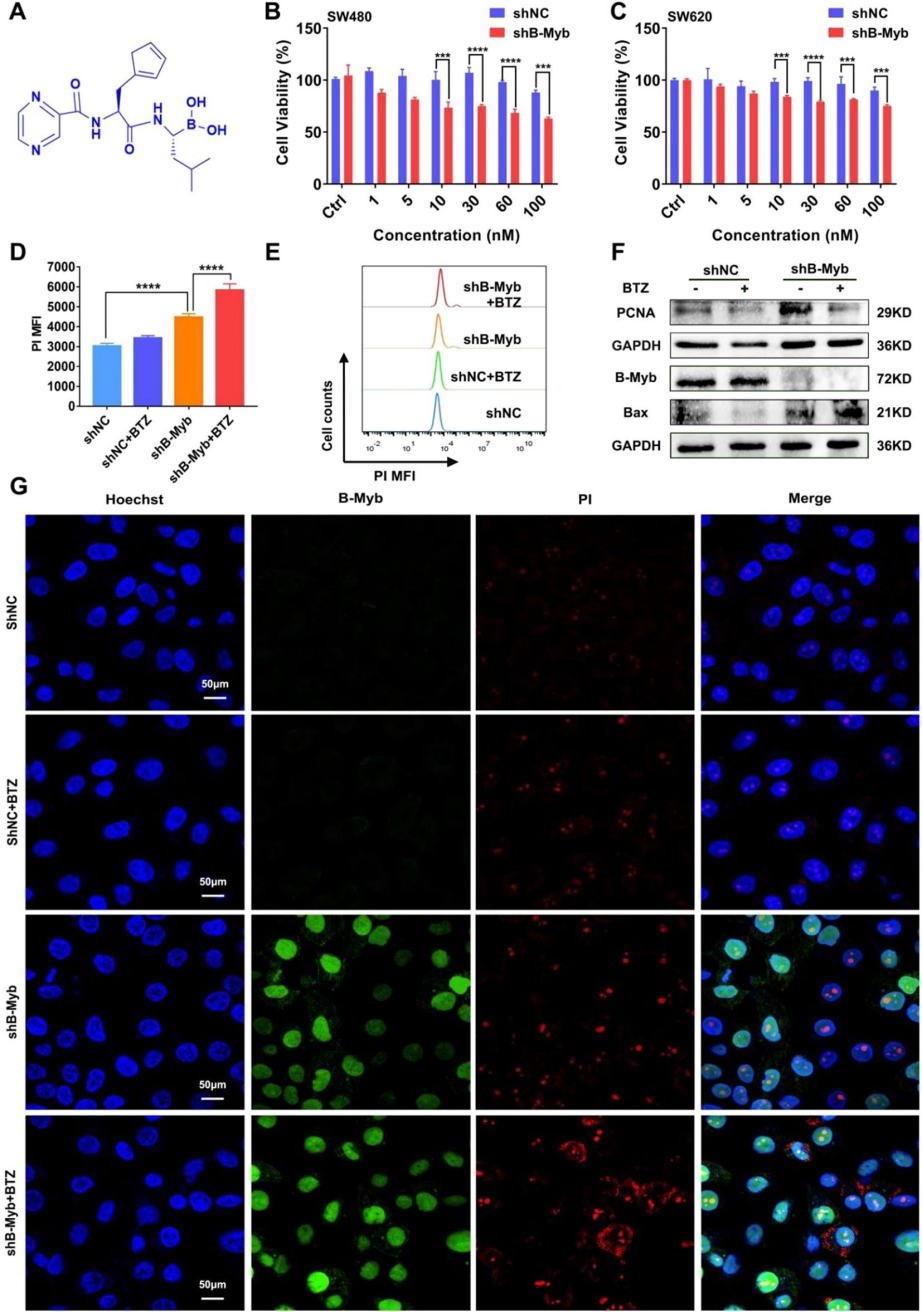
Bortezomib further boosted apoptosis in colorectal cancer cells of B-Myb stable knockdown. The colorectal cancer cells (SW480 or SW620) were treated by BTZ for 24 h. (**A**) The chemical structure of bortezomib was presented. (**B–C**) CCK-8 assay indicated BTZ significantly suppressed the cell viability in SW480 cells (**B**) and SW620 cells of B-Myb (**C**) deletion when the concentration reached to 30 nM in response to different concentrations treatment (1, 5, 10, 30, 60, and 100 nM). Values were means ± SD (n = 3, ***p < 0.001, ****p < 0.0001). (**D–E**) Cellular apoptosis rate was assayed using PI staining. The PI positive cells were calculated by flow cytometry. Geometric means were applied to quantify the MFI. Values were means ± SD (n = 3, **** *p* < 0.0001). (**F**) The molecules of B-Myb, proliferation and apoptosis (PCNA, Bax) were measured by WB. (**G**) The PI positive cells were also observed by laser confocal microscopy.

**Figure 3:**
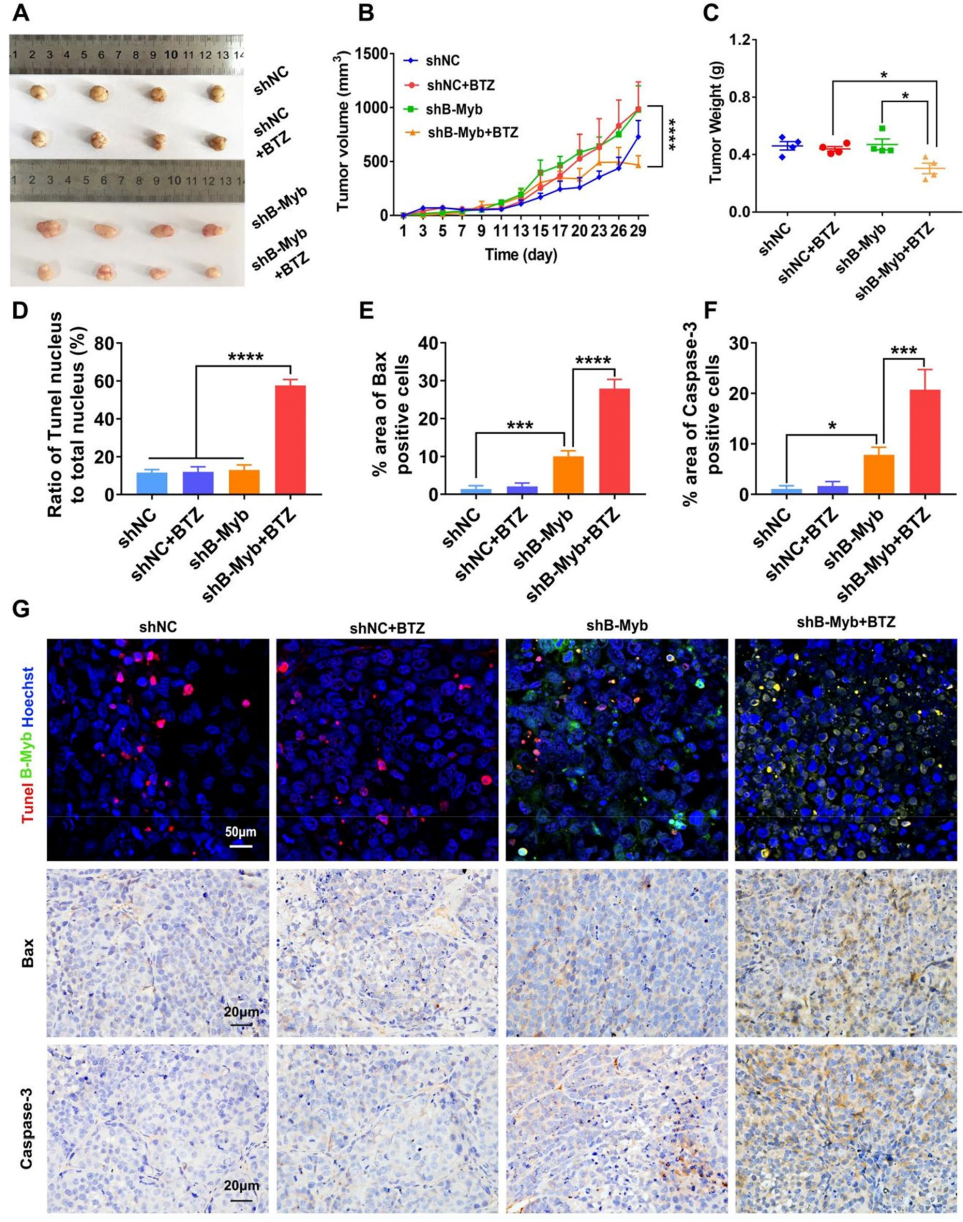
Bortezomib showed excellent anti-cancer efficacy in B-Myb knockdown colorectal cancer cell-bearing mice. BTZ was administered every three days for a total of 5 administrations (i.p.). The tumor grafts were harvested upon the end of treatment. (**A**) The tumor grafts were photographed. (**B**) The tumor volume was monitored during the treatment. (**C**) The tumor tissues were weighed after the extraction of tumor grafts. (**D–G**) IHC and TUNEL staining were utilized to evaluate cancer cells’ apoptosis, including Bax and Caspase-3 expression (**G**), which was quantified (**D**–**F**). Values were means ± SD (n = 4, *p < 0.05,****p < 0.0001).

To further understand whether the deletion of B-Myb could enhance the efficacy of chemotherapeutic agents, we proceeded with the following work using BTZ (Fig. 2A). Cell viability assay showed that the viability of the control colorectal cancer cells varied little in response to BTZ at the concentrations of 1–100 nM. However, BTZ treatment significantly inhibited the cell viability in B-Myb–defective colorectal cancer cells of SW480 and SW620 when the concentration reached to 30 nM (Fig. 2B–C). Therefore, the concentration of 30 nM was preferred for following experiments of BTZ treatment. In agreement with the results of cell viability, the findings of apoptosis assay also indicated that BTZ had a pronounced anticancer effect on colorectal cancer cells in the absence of B-Myb (Fig. 2D–E). Additionally, the expression of an apoptosis-associated protein Bax was enhanced, whereas the expression of a proliferation-associated molecule PCNA was reduced by BTZ in B-Myb–deleted colorectal cancer cells (Fig. 2F). As shown in Fig. 2G, there was increased PI fluorescence in BTZ-treated shB-Myb cells, consistent with the quantitative assays in Fig. 2D–E. The in vivo relevance of the in vitro findings was validated on tumor-bearing mice. We found a significant suppression of the tumor grafts in BTZ-treated shB-Myb colorectal cancer cell–bearing mice, as evidenced by the reduced volume and weight of the tumor grafts (Fig. 3A–C). In contrast, BTZ showed almost no efficacy in the control groups (Fig. 3A–C). Notably, the B-Myb–defective cancer cells showed more pronounced apoptosis in the presence of BTZ, as evidenced by the stronger expression of Bax and Caspase-3 and more pronounced TUNEL staining (Fig. 3G), which was quantified as presented in Fig. 3D–F. Furthermore, the body weight of the tumor-bearing mice varied little during BTZ treatment and there were few morphological changes in vital organs, suggesting that BTZ did not exert cytotoxicity on normal cells (Figs. S5, S6). Taken together, these data strongly indicate that BTZ has prominent anticancer efficacy in B-Myb–defective colorectal cancer.

### BTZ promotes DNA damage in B-Myb–defective colorectal cancer

To reveal the mechanism by which BTZ affects B-Myb–deficient colorectal cancer, RNA sequencing (RNA-Seq) was utilized to determine the differential gene expression between the shB-Myb and shB-Myb-BTZ groups. The differentially expressed genes (DEGs) were identified, including 2019 upregulated and 1804 downregulated genes, as presented by the volcano plots in Fig. S7. Accordingly, the Kyoto Encyclopedia of Genes and Genomes (KEGG) functional enrichment analysis and pathway classification were performed. We found that the protein processes in the endoplasmic reticulum (ER), p53 signaling pathway were among the top 20 pathways in the shB-Myb-BTZ group compared with the shB-Myb group (Fig. S7). As reported, p53 activation elicits the downstream signaling pathway of DNA damage and cell cycle arrest in cancer cells, which may be an essential contributor to the enhanced efficacy of BTZ^27–30^.

**Figure 4:**
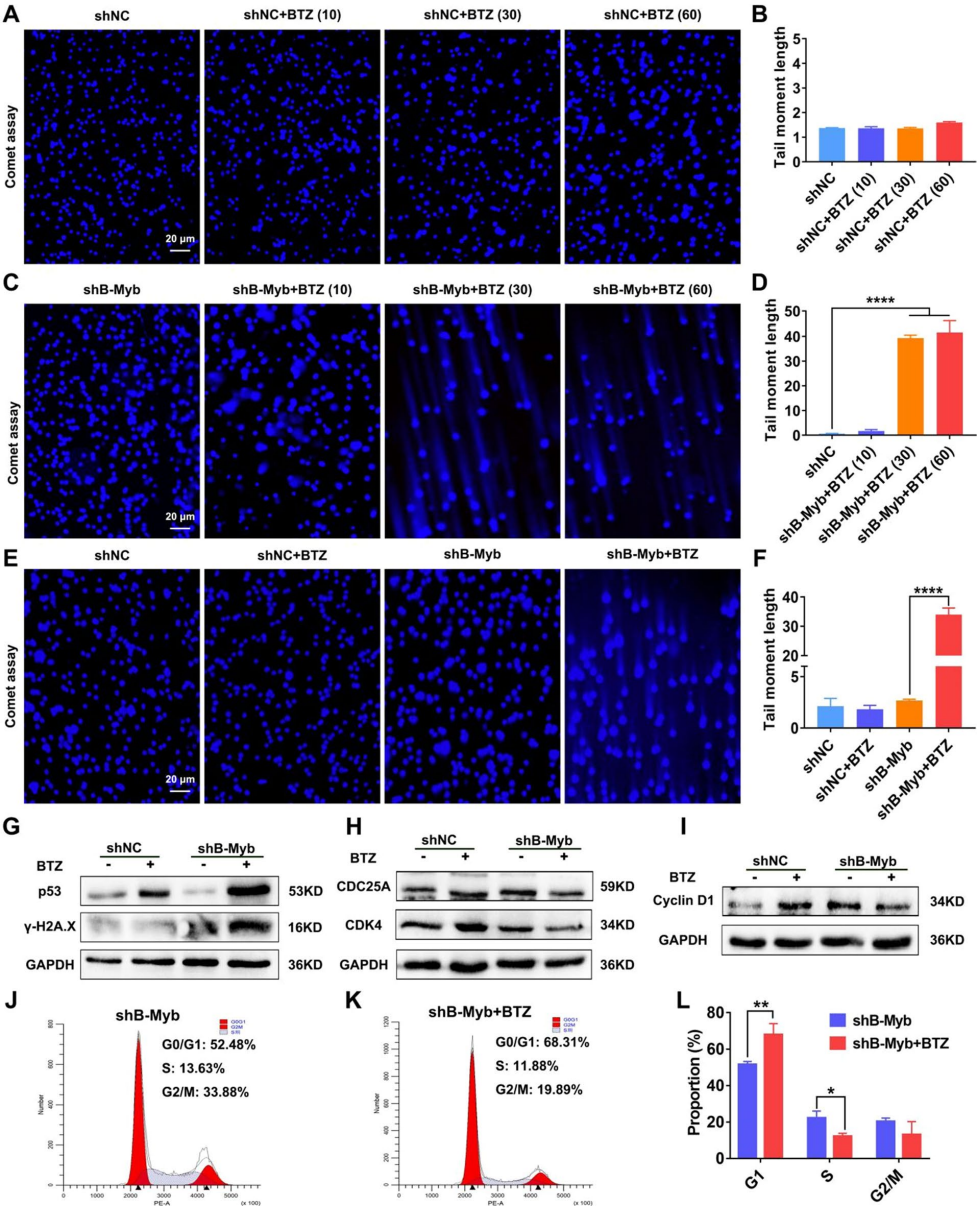
BTZ activated DNA damage in B-Myb defective colorectal cancer cells. The cancer cells were treated by BTZ for 24 h. (**A–D**) The DNA double-strand break of cells at different BTZ concentrations (10, 30, and 60 nM) was observed. (**E–F**) The DNA double-strand break was assayed by single-cell gel electrophoresis. The length of the comet tail was assayed. (**G**) The p53, γ-H2A.X molecules were detected using WB, suggesting prominent DNA damage in B-Myb defective SW480 cells treated by BTZ. (**H–I**) The expression of CDK4, CyclinD1 and CDC25A, which manipulate cell cycle progression, was investigated using WB. The mean gray was quantitatively assayed. (**J–L**) The cell cycle was assayed by PI staining and flow cytometry. The G1, S and G2/M phases were calculated. Geometric means were used to quantify the MFI. Values were means ± SD (n = 3, *p < 0.05, **p < 0.01, ***p < 0.001, ****p < 0.0001).

**Figure 5:**
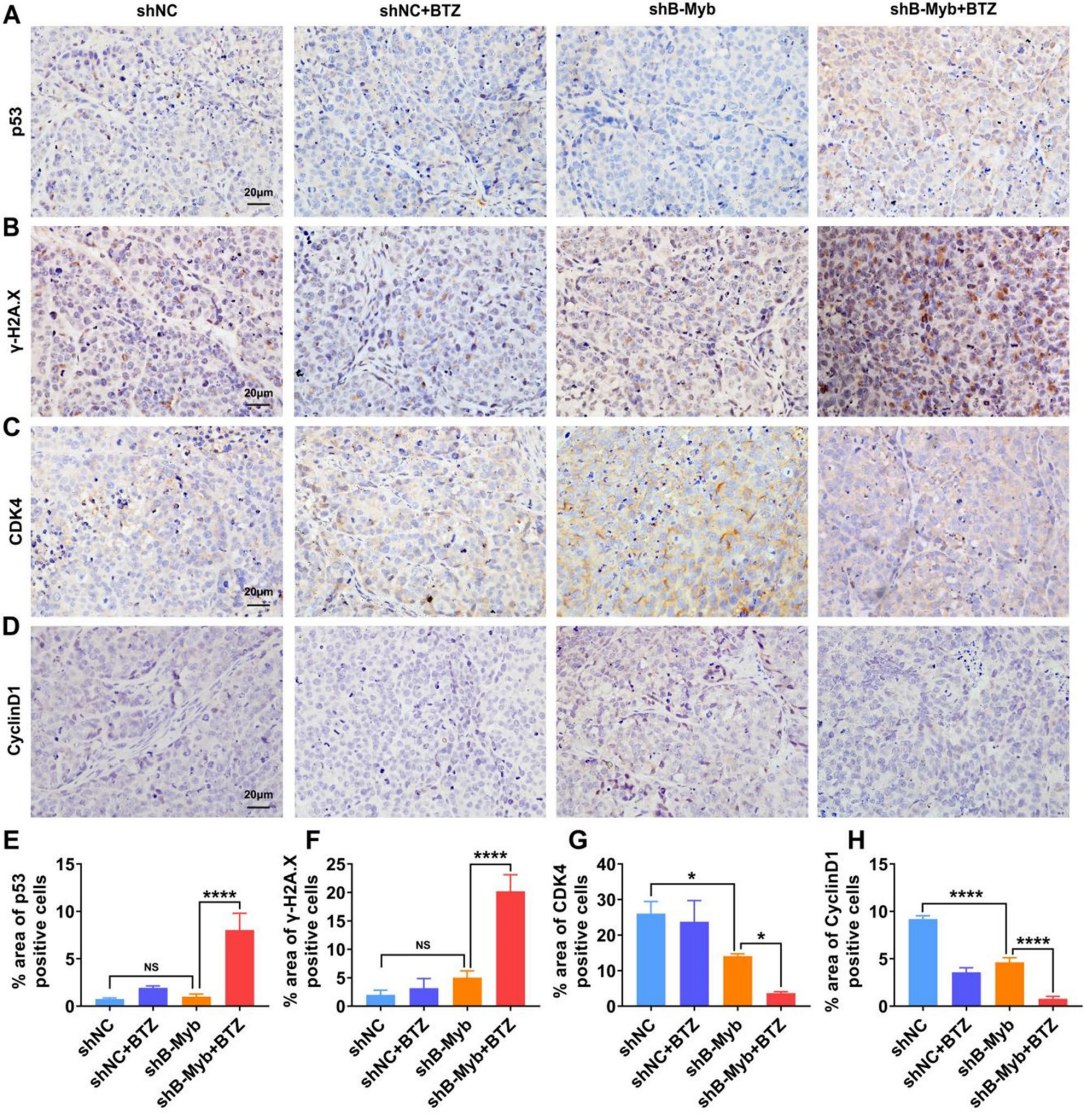
Prominent DNA damage was observed in BTZ-treated shB-Myb SW480 cell-bearing mice. (**A–B**) IHC staining evidenced BTZ treatment induced significant DNA damage in tumor-bearing mice of B-Myb deletion, as characterized by enhanced expression of p53 and γ-H2A.X. (**C–D**) The proteins controlling cell cycle (CDK4 and CyclinD1) in tumor grafts were assayed by IHC. (**E–H**) The area of positive cells were calculated. Values were means ± SD (n = 4, *p < 0.05, **p < 0.01, ***p < 0.001, ****p < 0.0001).

Therefore, we further explored the mechanism from the perspective of DNA damage and cell cycle blockage. The DNA damage of the cells also showed a concentration dependence in response to BTZ treatment, with significant DNA damage commencing at 30 nM in the shB-Myb group (Fig. 4A–D). Thus, the concentration of 30 nM was preferred for all subsequent studies of BTZ treatment. As depicted in Fig. 4G, compared with the shNC group, BTZ induced stronger p53 and γ-H2A.X expression in colorectal cancer cells with B-Myb deletion, confirming that prominent DNA damage appeared in the absence of B-Myb. Comet assay demonstrated that BTZ treatment triggered prominent DDSB in B-Myb–defective colorectal cancer cells (Fig. 4E–F). To validate the in vivo relevance of the in vitro findings, we also analyzed the expression of p53 and γ-H2A.X in the tumor tissues of the mice treated with BTZ. As shown in Fig. 5A–B, the IHC analysis demonstrated that BTZ dramatically damaged the DNA of the cancer cells in the tumor grafts devoid of B-Myb, which was quantified (Fig. 5E–F). Hence, these findings strongly suggest that BTZ leads to serious DNA damage in B-Myb–defective colorectal cancer.

In addition to the investigation of DNA damage, we proceeded to analyze the cell cycle in the shNC and shB-Myb cells exposed to BTZ. The rationale was based on the fact that severe DNA damage (e.g., DDSB) in cancer cells that cannot be repaired would cause cell cycle arrest. Not surprisingly, the progression from the G1 phase to the S phase was arrested in the response to BTZ in shB-Myb SW480 cells, as indicated by the decreased expression of CDC25A, CDK4, and CydlinD1 (Fig. 4H–I). These molecules are downstream in the signaling of DNA damage, which could further regulate the cell cycle. As expected, the direct findings of the cell cycle assay proved that BTZ treatment blocked the G1 phase of the cell cycle (Fig. 4J–L). Accordingly, the molecules involved in the cell cycle (CDK4 and CyclinD1) were downregulated in shB-Myb-SW480 cell–bearing mice that received BTZ treatment (Fig. 5C–D, G–H). Hence, we showed a critical role of cell cycle arrest in BTZ efficacy when B-Myb was stably knocked down. Altogether, BTZ promotes critical DNA damage and cell cycle arrest in B-Myb–defective colorectal cancer, further exhibiting the anticancer effect.

### Recovery of B-Myb in B-Myb–deficient colorectal cancer cells attenuates BTZ-induced DNA damage and apoptosis

**Figure 6:**
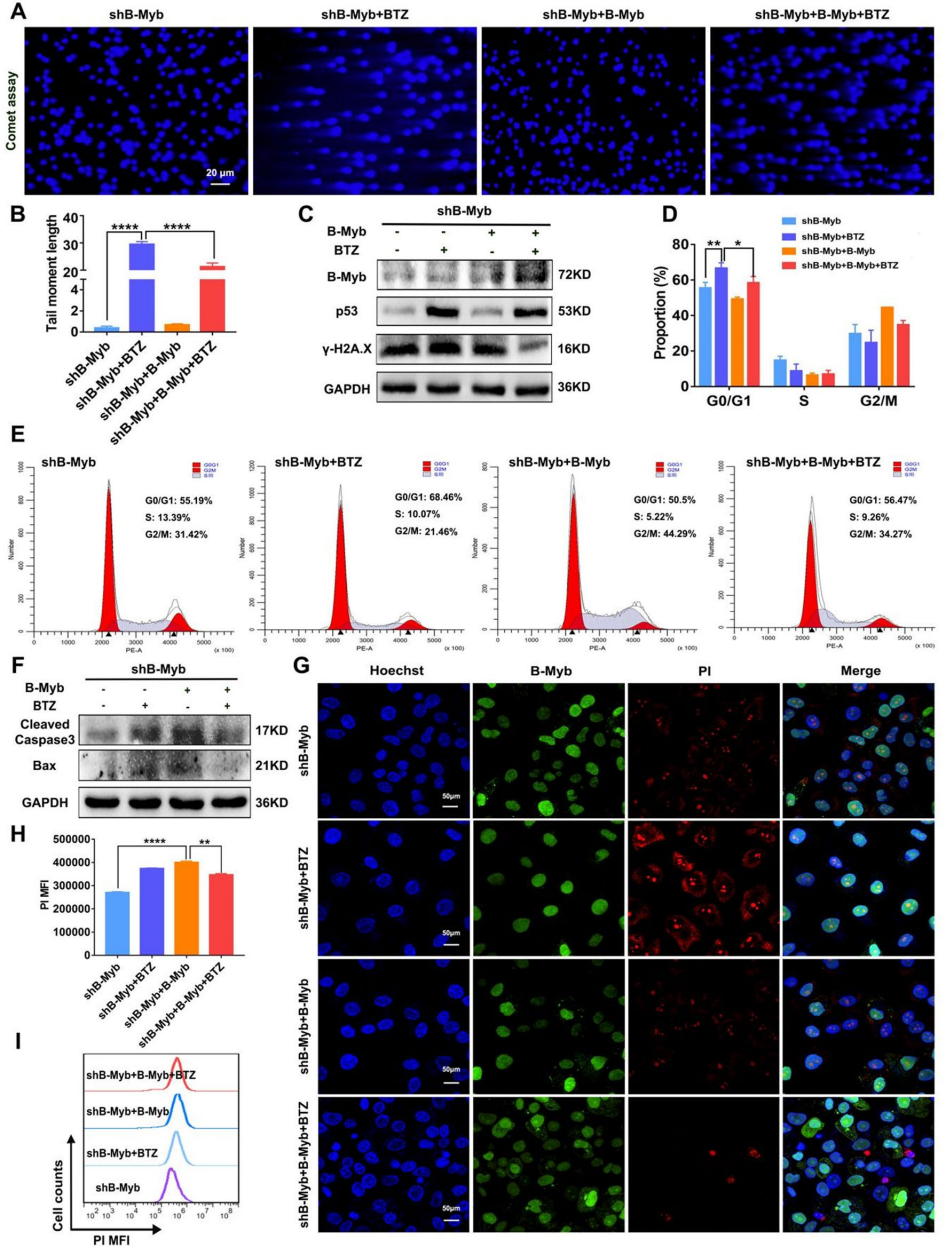
Over-expression of B-Myb reduced the DNA damage, cell cycle arrest, and apoptosis induced by BTZ in shB-Myb SW480 cells. The shB-Myb SW480 cells were transfected with B-Myb plasmid, then treated by BTZ. (**A–B**) Comet experiments were applied to assay the DNA double-strand breakage. The length of the comet tail was analyzed. (**C**) The expression of B-Myb, p53 and γ-H2A.X was assayed using WB. (**D–E**) The cell cycle was analyzed by flow cytometry. (**F**) Apoptosis-associated molecules Casepase-3 and Bax were measured using WB. (**G–I**) The apoptosis of cells was measured by PI staining. The positive cells were observed by laser confocal microscopy (**G**) and flow cytometry (**H**–**I**). Geometric means were used to quantify the MFI. Values were means ± SD (n = 3, *p < 0.05, **p < 0.01, ***p < 0.001, ****p < 0.0001).

To provide an in-depth confirmation of the critical role of B-Myb in the therapeutic effect of BTZ by inducing DNA damage and cell cycle arrest, B-Myb was overexpressed in B-Myb–defective SW480 cells for further experiments. As expected, we first confirmed the results by showing that the overexpression of B-Myb in shB-Myb SW480 cells abated BTZ-introduced elevated expression of p53 and γ-H2A.X (Fig. 6C). Moreover, the DDSB was weakened in the shB-Myb SW480 cells when BTZ and B-Myb–overexpressing plasmid were added together (Fig. 6A–B). The flow cytometry results revealed that overexpression of B-Myb impaired BTZ-mediated arrest of the cell cycle in B-Myb–defective colorectal cancer cells (Fig. 6D–E). These data suggest that the recovery of B-Myb attenuates BTZ-triggered DNA damage and cell cycle arrest in B-Myb–deficient colorectal cancer cells.

Subsequently, we examined the anticancer effect induced by BTZ and B-Myb–overexpressing plasmid in shB-Myb SW480 cells. In agreement with the results of DNA damage and cell cycle, the increased apoptosis level of the shB-Myb SW480 cells treated with BTZ was slightly dampened after B-Myb overexpression, as revealed by the suppressed expression of Bax and Cleaved-Caspase-3 (Fig. 6F). Similarly, PI staining showed that the fluorescence was marginally weakened in the presence of B-Myb–overexpressing plasmid, which was quantified as well (Fig. 6G–I). Actually, in colorectal cancer cells, there must be more than one molecule that affects the anticancer efficacy of BTZ. The observed effect of B-Myb recovery on attenuating the efficacy of BTZ was statistically significant but not particularly strong. In summary, recovery of B-Myb in B-Myb–missing colorectal cancer cells attenuates BTZ-induced anticancer efficacy.

### BTZ improves immunogenicity and drives macrophage activation in case of B-Myb deficiency

In general, damage to cell nuclear DNA leads to increased release of DAMPs, which prompts enhanced immunogenicity of cancer cells^31,32^. The expression of classical nuclear DAMPs, including HMGB1 and HSP90, was upregulated in BTZ-treated colorectal cancer cells and more significantly in B-Myb–deficient cells in vitro and in vivo (Fig. S8). Enhanced immunogenicity in tumor tissues should lead to more immunocyte infiltration as well as activation, thereby driving an antitumor immune response^33^.

**Figure 7:**
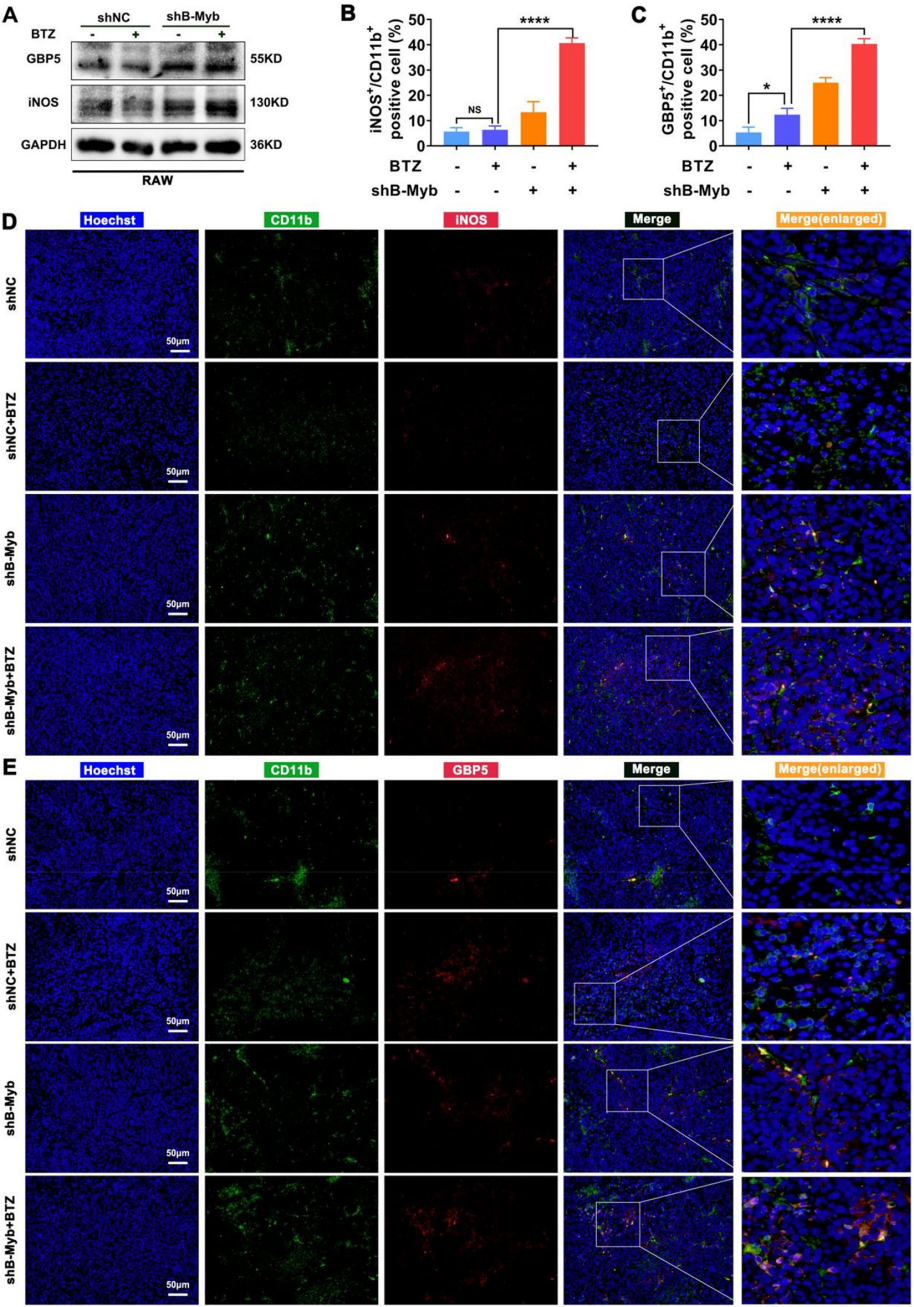
Type-I macrophages were accumulated in B-Myb defective colorectal cancer in the presence of BTZ. (**A**) The expression of GBP5 and iNOS, which are biomarkers of M1, was detected by WB. (**B–E**) Multiple immunofluorescence staining revealed the macrophages’ infiltration (expression of CD11b) and M1 polarization (iNOS and GBP5). Blue fluorescence came from the nucleus. Green fluorescence came from CD11b. Red fluorescence came from iNOS (**D**) or GBP5 (**E**). The CD11b/iNOS positive cells and CD11b/BGP5 positive cells were quantified (**B**–**C**). Values were means ± SD (n = 4, **p < 0.01, ***p < 0.001).

Hence, the enrichment and phenotype of macrophages, which are the main immune cells in B-Myb–deficient colorectal cancer treated by BTZ, were analyzed. Although lymphocytes were reduced in the BALB/c-nu mice, macrophage infiltration in the tumor grafts was not affected. As expected, the expression levels of iNOS and GBP5, which are biomarkers of M1 macrophages, were upregulated in response to the conditional medium from BTZ-treated shB-Myb colorectal cancer cells (Fig. 7A). In addition, M1 macrophages, which are considered antineoplastic, became more numerous in the BTZ-treated shB-Myb colorectal cancer cells–bearing mice, as evidenced by the increased CD11b/iNOS and CD11b/GBP5 double-positive cells, which was quantified (Fig. 7B–E). These findings strongly suggest that BTZ improves immunogenicity and further drives immunocytes activation when B-Myb is deficient, which may also be an important explanation for the better therapeutic effect in vivo as shown in Fig. 3.

## Discussion and conclusion

**Figure 8:**
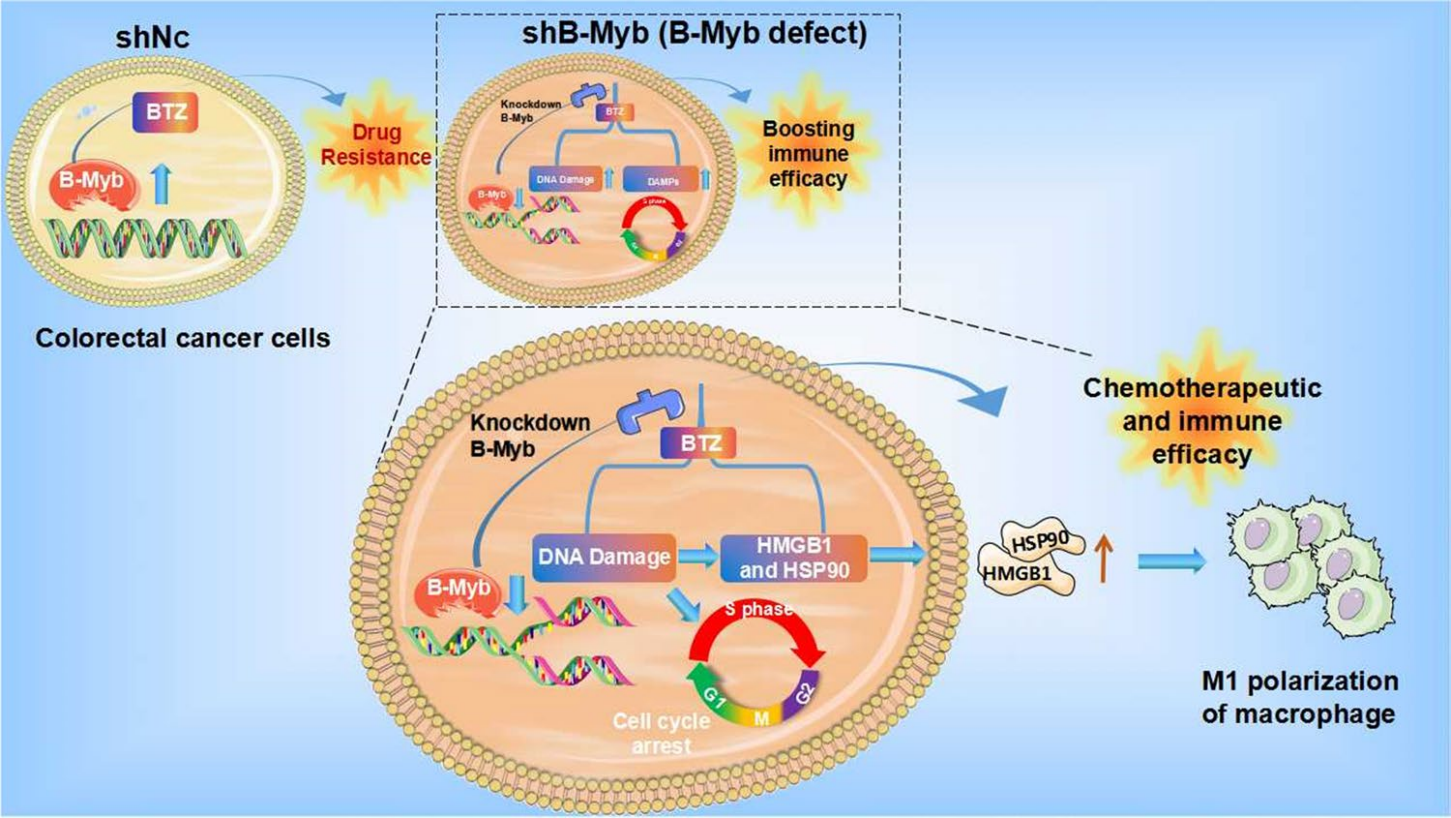
The schematic diagram of the current work. High expression of B-Myb leads to DNA repair and drug resistance in colorectal cancer. BTZ could promote cell apoptosis in B-Myb defective tumors, wherein pronounced DNA damage and cell cycle arrest were induced. The immunogenic cell death and macrophage M1 polarization were observed thereupon. Recovery of B-Myb attenuates BTZ-introduced DNA damage and blockage of the cell cycle, thereby attenuating the anti-cancer effect.

We showed that B-Myb acts as a contributor to anti-DNA damage and immunogenic death to affect drug resistance to BTZ in colorectal cancer. BTZ accelerates DNA damage and thereby enhances the immunogenicity of colorectal malignant cells in the absence of B-Myb, resulting in better antitumor immune efficacy of BTZ (Fig. 8).

In the present study, we further demonstrated that the knockdown of B-Myb significantly augmented the level of apoptosis in colorectal cancer cells (Fig. 1). It has been shown that B-Myb is highly overexpressed in colorectal cancer, wherein it promotes cancer progression by interacting with E2F2^13^. A plausible explanation is that colorectal cancer cells with high expression of B-Myb could promote the expression of certain antiapoptotic molecules at the transcriptional level, thereby enabling cancer cells to resist aging and drug-induced DNA damage. This is a major contributor to the enhanced sensitivity of colorectal cancer cells to chemotherapeutic agents after the knockdown of B-Myb. Therefore, the present research worked on the effect of B-Myb deficiency on the BTZ’s anticancer efficacy. As reported, A-Myb has important functions in spermatogenesis and mammary gland development^34,35^, whilst C-Myb regulates the differentiation of hematopoietic stem cells and correlates with lymphoma^36,37^. Distinct from A-Myb and C-Myb, the high expression of the B-Myb gene, principally in malignantly proliferating cells, dictates that B-Myb has a significant role in tumor development^17–19^. Consistently, the present study also confirmed that the knockdown of B-Myb in colorectal cancer cells increased BTZ’s efficacy as displayed in Figs. 2, 3.

The mechanism of action of BTZ is based on the inhibition of the proteasome, which in turn inhibits the degradation of certain intracellular proteins and indirectly promotes their intracellular accumulation^7,38,39^. BTZ is a synthetic di-peptide boronic acid analog, which belongs to a proteasome inhibitor, with low toxicity and side effects due to its ability to regulate protein degradation within malignant cells. BTZ has higher therapeutic efficacy and longer survival for leukemia patients compared with other traditional chemotherapeutic agents. In addition, combining BTZ with various chemotherapeutic agents is also clinically significant^40,41^. The present study did not explore the reasons for the better efficacy of BTZ in B-Myb–deficient colorectal cancer in terms of proteasome inhibition and protein ubiquitination, which is a limitation of the present work. However, we discovered BTZ-mediated enhanced DNA damage and immunogenicity when B-Myb was absent, wherein BTZ could block the cell cycle more effectively in B-Myb–deficient colorectal cancer and induce intense DNA damage (Figs. 4, 5), which has not been reported before. This clearly explains why cancer cells with high expression of B-Myb are resistant to BTZ. In addition, some studies have shown that BTZ can enhance the immunogenicity of cancer cells^42,43^. Several recent reports have also revealed a great correlation between B-Myb and immunocyte infiltration^44^. Interestingly, the present work showed that HMGB1 and HSP90, classical immunogenic molecules, were upregulated in response to BTZ in B-Myb–deficient colorectal cancer cells, leading to macrophage M1 polarization (Figs. 7 and S8), which demonstrated an important relationship between B-Myb and immune microenvironment activation. Consequently, B-Myb may be a novel target for drug immunotherapy in the future.

Collectively, B-Myb deletion in colorectal cancer facilitates the immunogenic death of malignant cells, thereby further rendering the immune efficacy of BTZ by amplifying DNA damage, promoting immunogenic cell death, and arresting the cell cycle (Fig. 8). The present study provides an effective molecular target for colorectal cancer immunotherapy with BTZ.

### Supplementary Information

Supplementary Figures.

## Data Availability

The datasets used and/or analyzed during the current study are available from the corresponding author upon request.

## Abbreviations

BTZ: Bortezomib
CRC: Colorectal cancer
COAD: Colon adenocarcinoma
READ: Rectum adenocarcinoma
CDK: Cycle-dependant kinase
DAMPs: Damage associated-molecular patterns
DDSB: DNA double-strand break
GBP5: Guanylate-binding protein 5
HMGB1: High mobility group box 1
HSP90: Heat shock protein 90
ICD: Immunogenic cell death
IHC: Immunohistochemistry
iNOS: Induction of inducible nitric oxide synthase
WB: Western blotting
PI: PropidiumIodide
MFI: Mean fluorescence intensity
B-Myb: MYB proto-oncogene like 2
TAM: Tumor associated macrophages
