# Supplementary material for: B-Myb deficiency boosts bortezomib-induced immunogenic cell death in colorectal cancer
Source: Sci Rep. 2024 Apr 2;14:7733. doi: 10.1038/s41598-024-58424-w (PMC10987531; doi:10.1038/s41598-024-58424-w)
Supplement: Supplementary file 1 — Supplementary Figures. [file 41598_2024_58424_MOESM1_ESM.pdf]

Supplementary Materials

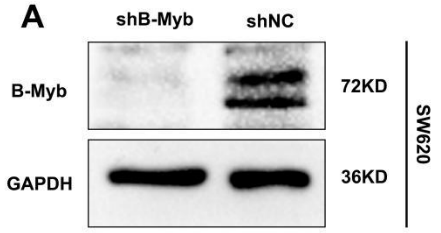

Figs. S1. The expression of B-Myb in SW620 cells and the lentiviral shRNA-treated cells.

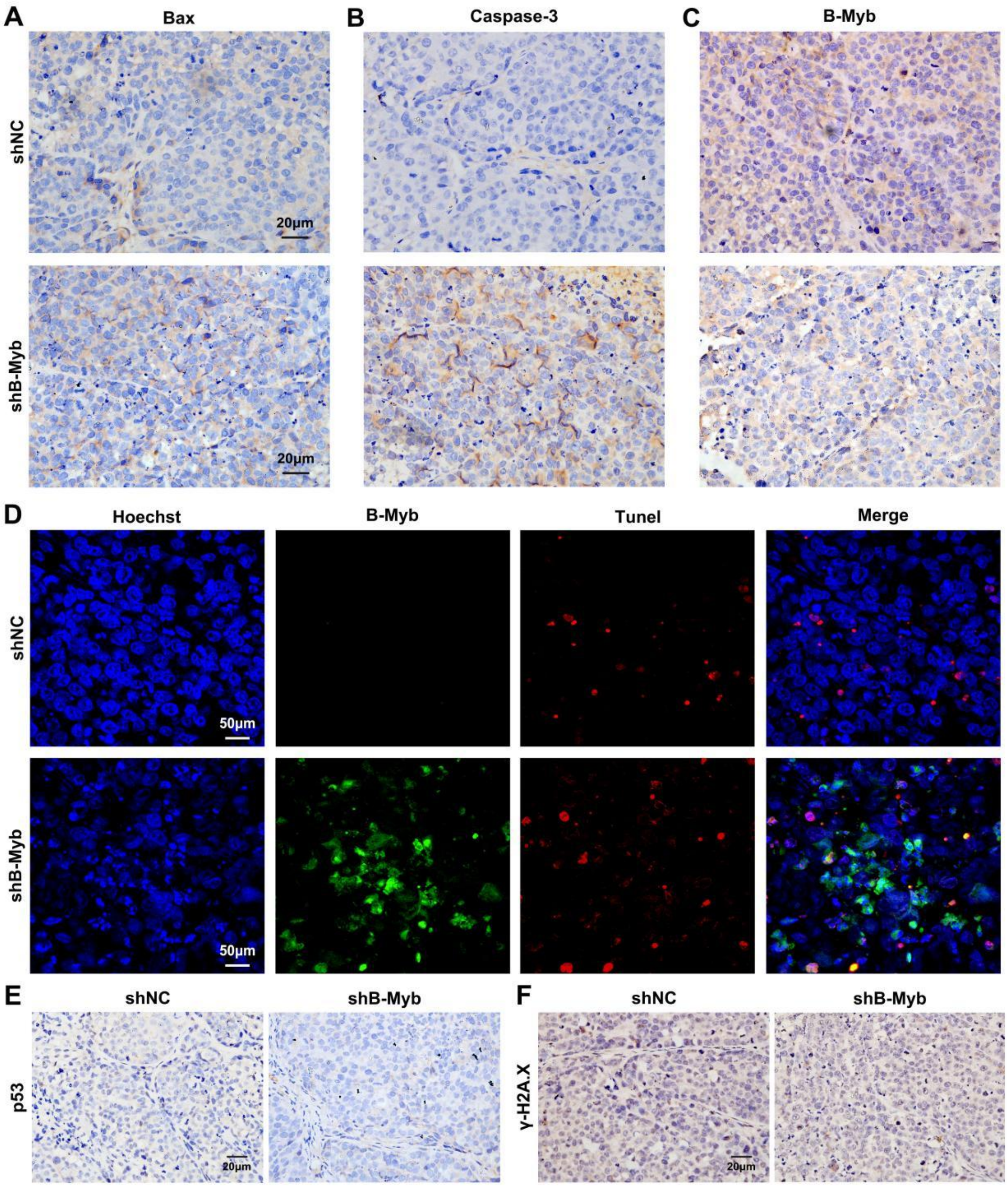

Figs. S2. Apoptosis and DNA damage-associated biomarkers were detected by IHC assay and Tunel staining in tumor grafts of B-Myb knockdown colorectal cancer cells-bearing mice. The tumor grafts from SW480 cell-bearing mice were extracted. **A-C:** The expression of Bax, Caspase-3 and B-Myb in tumor tissues was analyzed using IHC. **D**: Tunel staining was applied to assay the apoptosis of malignant cells in tumor grafts. **E-F**: IHC assay revealed the DNA damage-associated molecules (p53 and γ-H2A.X) expression.

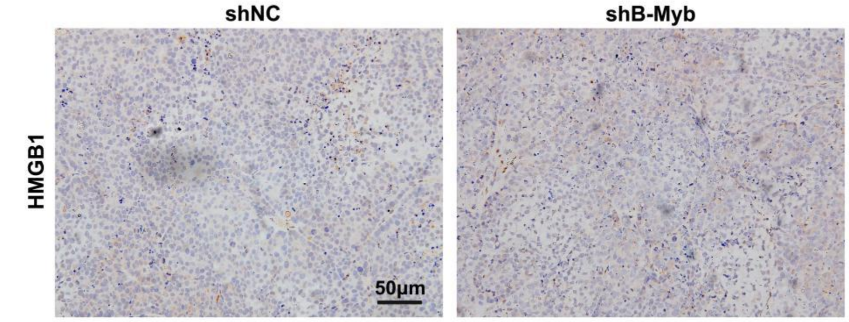

Figs. S3. HMGB1 expression in tumor grafts of B-Myb knockdown colorectal cancer cells-bearing mice was detected by IHC assay.

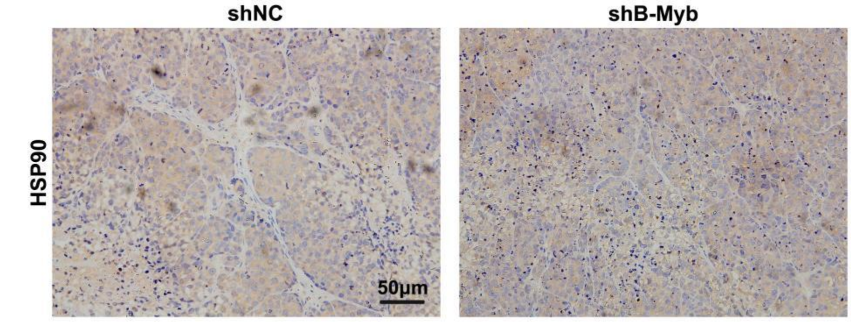

Figs. S4. HSP90 expression in tumor grafts of B-Myb knockdown colorectal cancer cells-bearing mice was detected by IHC assay.

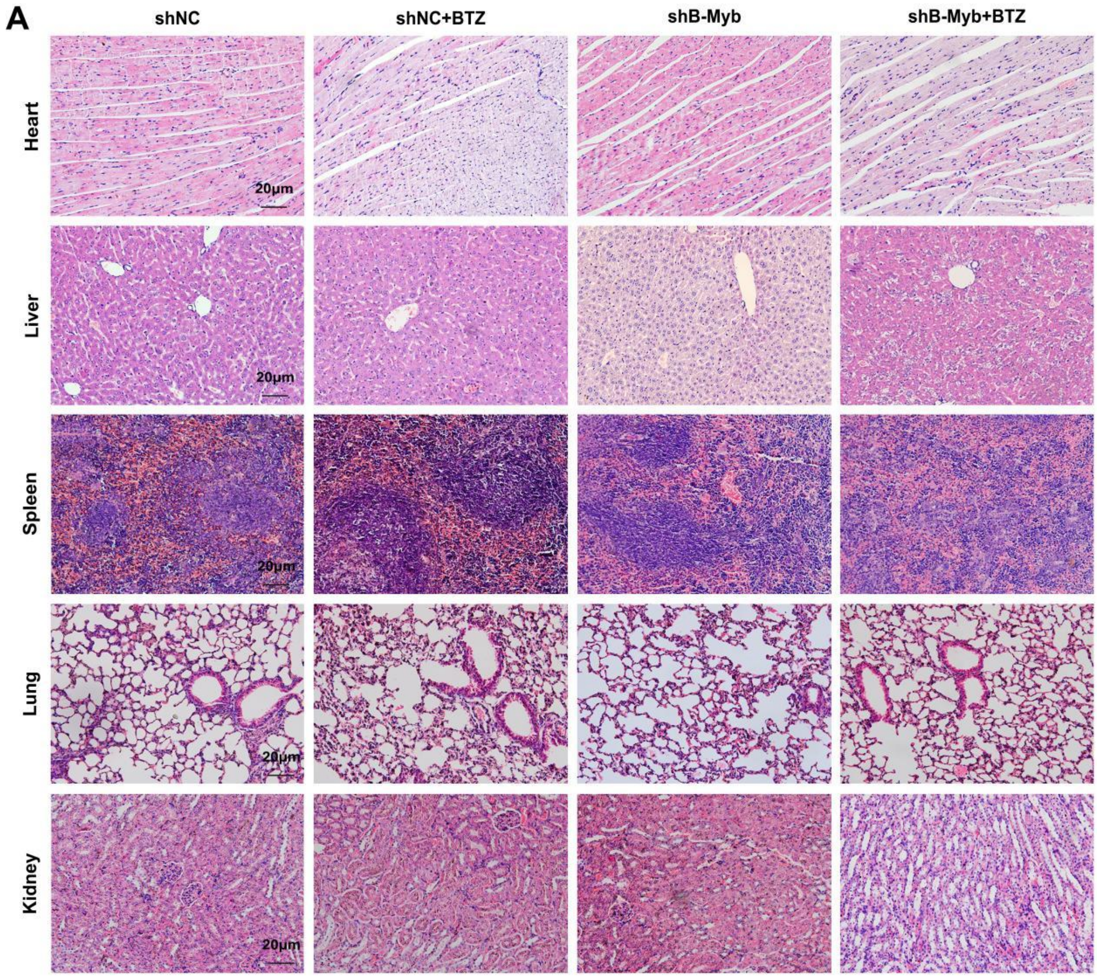

Figs. S5. The morphological properties of vital organs was detected by HE staining.

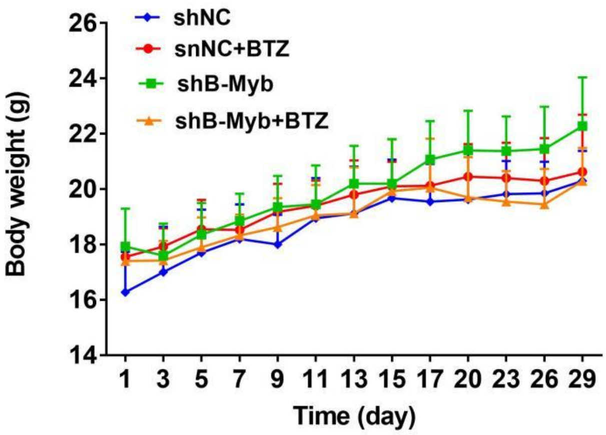

Figs. S6. The body weight was monitored during the bortezomib treatment.

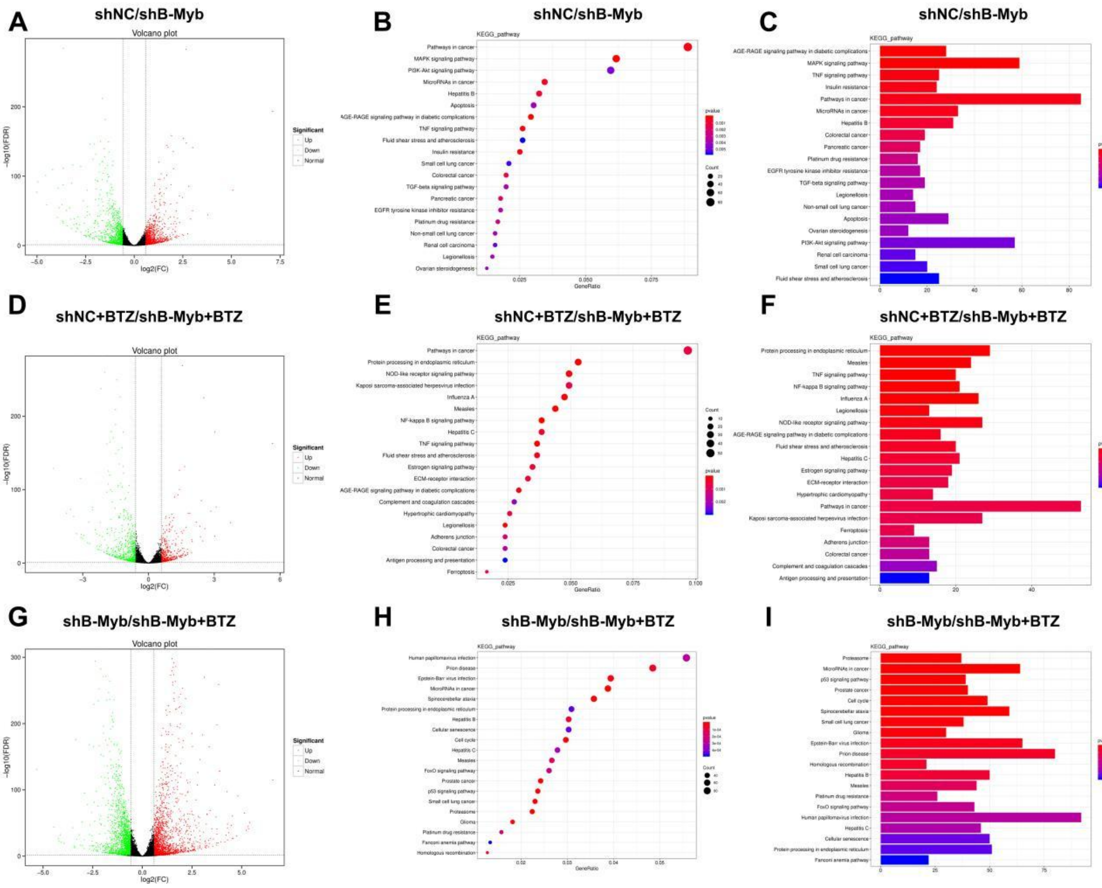

Figs. S7. The differential expressed genes (DEGs) and enrichment between shNC and shB-Myb groups (A-C), shNC-BTZ and shB-Myb-BTZ groups (D-F), shB-Myb and shB-Myb-BTZ groups (G-I) were analyzed using the RNA-Seq technique, wherein the KEGG signaling pathway enrichment of DEGs was assayed and presented.

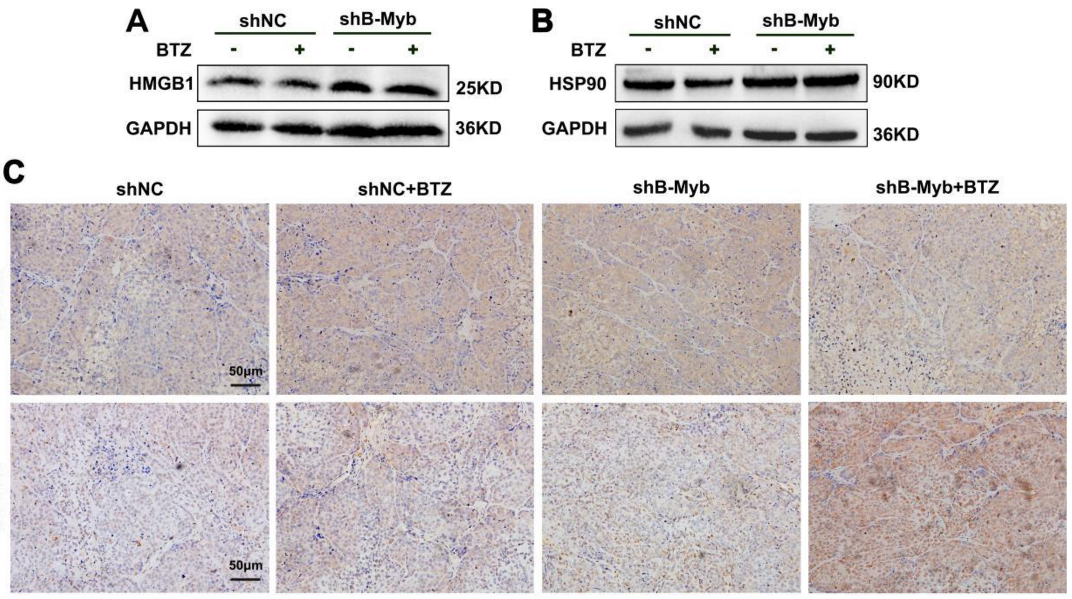

Figs. S8. WB and IHC assay was utilized to analyze the HMGB1 and HSP90 expression in colorectal cancer cells (A-B) and cancer tissue of tumor-bearing mice (C).

Original Figures for Blots in Figure 1

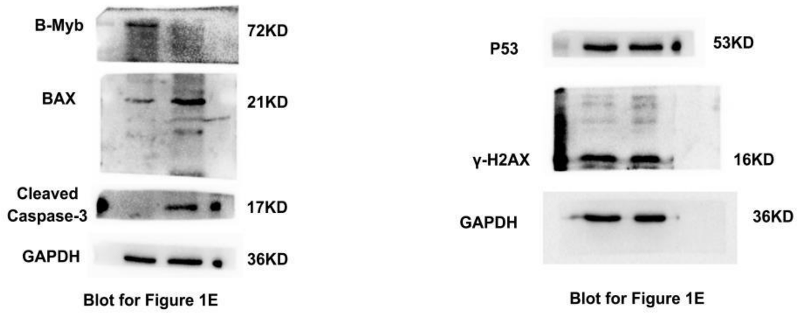

Original Figures for Blots in Figure 2

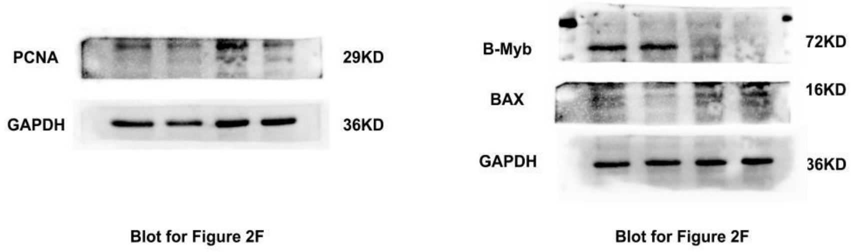

Original Figures for Blots in Figure 4

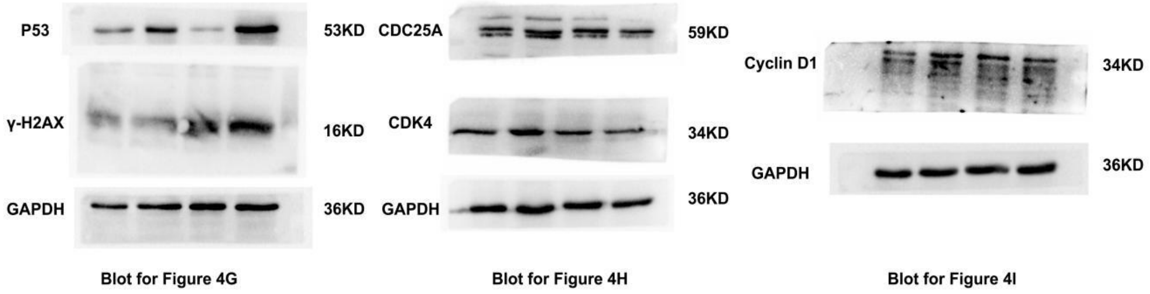

Figs. S9. The original data of western blots in the present work (Figs 1E, 2F, and 4G, H, I, images with membrane edges visible**).**

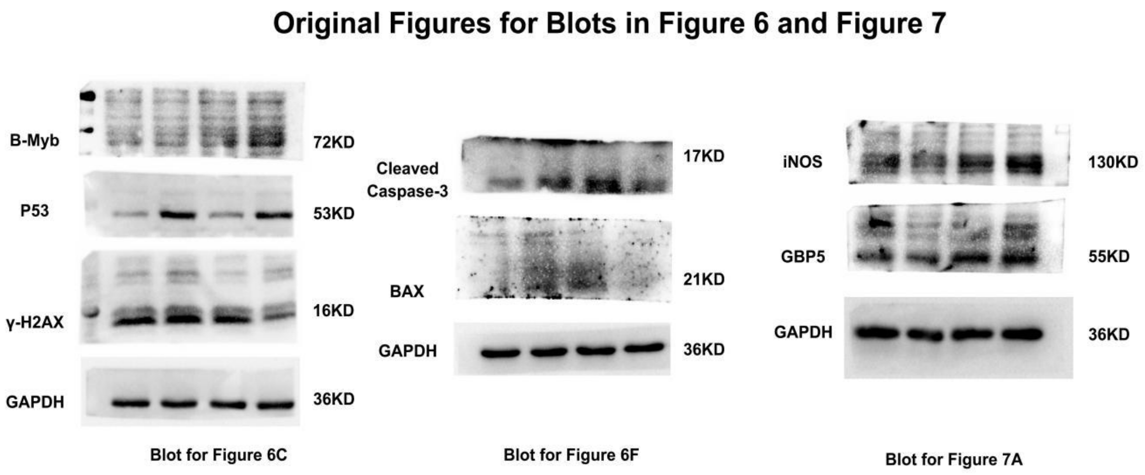

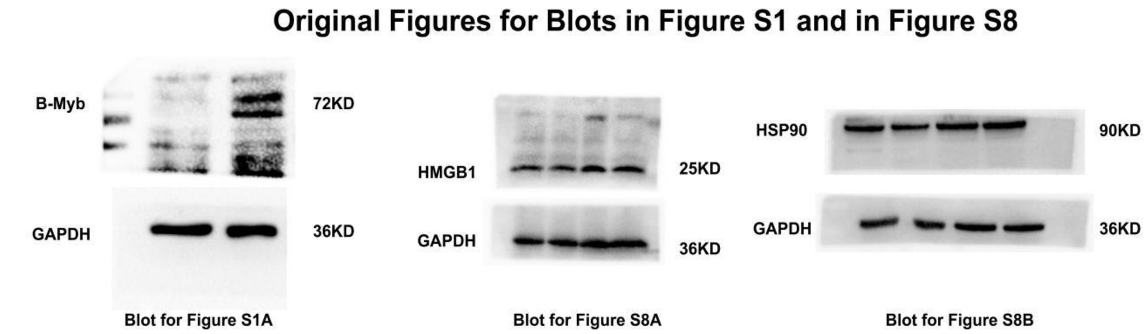

Figs. S10. The original data of western blots in the present work (Figs 6C, F, 7A, S1A, and S8A-B, images with membrane edges visible**).**
